# Proteomic signatures of cervical mucus associated with fertility in Bali heifers (*Bos javanicus*): Implications for biomarker-based selection in artificial insemination programs

**DOI:** 10.14202/vetworld.2026.135-148

**Published:** 2026-01-14

**Authors:** Muhammad Yusuf, Abdul Latief Toleng, Hasrin Hasrin, Abdullah Baharun, Athhar Manabi Diansyah, Santoso Santoso, Rahmat Rahmat, Andi Muhammad Alfian, Masturi Masturi, Sahiruddin Sahiruddin, Muhammad Fajar Amrullah, Ahmad Alfaruqi Syahrandi Adam, Miftahul Jannah

**Affiliations:** 1 Faculty of Animal Science, Hasanuddin University, Jl. Perintis Kemeredekaan Km. 10 Tamalanrea Makassar, South Sulawesi, Indonesia; 2 Faculty of Vocation, Hasanuddin University, Jl. Perintis Kemerdekaan Km. 10 Tamalanrea Makassar, South Sulawesi, Indonesia; 3 Faculty of Agriculture, Djuanda University, Jl. Tol Ciawi No. 1, Ciawi, West Java, Indonesia; 4 Research Center for Animal Husbandry, National Research and Innovation Agency, Cibinong Science Center, Bogor, Indonesia; 5 Faculty of Agriculture, Lambung Mangkurat University, Jl. Jenderal Ahmad Yani Km. 36, Banjarbaru, South Kalimantan, 70714, Indonesia; 6 Doctoral Program of Animal Biomedical Science, School of Veterinary Medicine and Biomedical Sciences, IPB University, Bogor, 16680, West Java, Indonesia; 7 Graduate School, Hasanuddin University, Jl. Perintis Kemeredekaan Km. 10 Tamalanrea Makassar, South Sulawesi, Indonesia; 8 Master Program of Animal Biomedical Science, School of Veterinary Medicine and Biomedical Sciences, IPB University, Bogor, 16680, West Java, Indonesia

**Keywords:** artificial insemination, Bali cattle, biomarkers, cervical mucus, fertility, heifers, proteomics, reproductive efficiency

## Abstract

**Background and Aim:**

Despite strong adaptive traits, the reproductive efficiency of Bali cattle (*Bos javanicus*) remains suboptimal, with low conception rates following artificial insemination (AI). Cervical mucus (CM) is a critical factor in sperm transport and fertilization; however, its molecular basis in relation to fertility has not been elucidated in this indigenous breed. This study aimed to characterize the proteomic profile of CM in Bali heifers and to identify protein biomarkers associated with fertility-related mucus quality.

**Materials and Methods:**

The study was conducted between February and August 2024 in South Sulawesi, Indonesia. Forty clinically healthy Bali heifers (2–3 years old) were sampled during natural oestrus and divided into good CM (GCM; n = 20) and poor CM (PCM; n = 20) groups using a validated five-parameter biophysical scoring system. CM proteins were extracted and analyzed using one-dimensional sodium dodecyl sulfate–polyacrylamide gel electrophoresis followed by liquid chromatography–tandem mass spectrometry. High-confidence protein identification was achieved at <1% false discovery rate, and differential abundance was evaluated using Benjamini–Hochberg correction (p < 0.05). Functional enrichment, correlation analysis with mucus traits, and receiver-operating-characteristic (ROC) analyses with cross-validation were performed.

**Results:**

Significant differences (p < 0.05) were observed between GCM and PCM groups for appearance, viscosity, spinnbarkeit, and ferning pattern, while pH did not differ. A total of 52 proteins were identified after quality control, of which 13 showed significant differential abundance. GCM was characterized by higher levels of NT5E, lactoferrin, SCGB1D, and lactotransferrin, whereas PCM showed enrichment of complement factor I (CFI), haptoglobin (HP), MUC5AC, FAIM2, TIMP2, PEBP4, SAA3, GRP, and IGL. Functional enrichment analysis indicated anti-inflammatory and epithelial-protective pathways in GCM, in contrast to complement activation, proteolysis, and oxidative remodeling in PCM. ROC analysis demonstrated excellent discriminative performance for NT5E (GCM) and CFI and haptoglobin (PCM), each achieving an area under the curve of 1.00 in this cohort.

**Conclusion:**

This study offers the first proteomic evidence connecting CM composition to fertility-related traits in Bali heifers. NT5E, CFI, and HP stand out as promising biomarkers for fertility screening, providing a molecular framework to improve AI efficiency and selection strategies in indigenous cattle.

## INTRODUCTION

Reproductive efficiency is a key factor in livestock productivity, especially for indigenous breeds, such as *Bos javanicus* (Bali cattle), which are crucial to Indonesia’s national meat self-sufficiency program. Although Bali cattle possess strong adaptive abilities and valuable genetic traits, their reproductive performance in the field remains suboptimal. Artificial insemination (AI), widely used to accelerate genetic improvement, has not achieved the expected results, with conception rates reported between 23% and 39%, significantly below the ≥60% threshold typically required for successful long-term breeding programs [1, 2].

One of the main constraints behind these low conception rates is the lack of molecularly informed methods for identifying reproductively capable females. Currently, selection decisions mainly rely on phenotypic criteria such as age, body condition score, and calving interval. While helpful on a descriptive level, these indicators do not reveal the underlying physiological and molecular mechanisms that control fertility. Consequently, selection for reproductive performance remains inconsistent and imprecise, which ultimately reduces the overall effectiveness of AI programs [3].

Recent advances in proteomic technologies offer a promising path for discovering fertility-related biomarkers at the molecular-level. Among female reproductive tract secretions, cervical mucus (CM) plays a key role in sperm transport, survival, and fertilization [4]. Its biochemical composition, especially its protein components, is dynamically altered by hormonal fluctuations throughout the estrous cycle. Glycoproteins such as mucins, along with immune-modulatory proteins, undergo significant changes in response to endocrine signals and are thought to be essential for creating an environment in the reproductive tract that supports successful fertilization [5, 6].

Proteomic profiling of CM, therefore, has significant potential to identify unique molecular signatures linked to fertile and subfertile phenotypes. However, existing research has mainly focused on male reproductive fluids, including seminal plasma and spermatozoa [7, 8], while studies on female reproductive secretions, especially in indigenous livestock populations in Indonesia, are still limited.

Although reproductive inefficiency in Bali cattle is well known, most research has focused on phenotypic, managerial, and hormonal factors affecting fertility, with limited attention to molecular processes in the female reproductive tract. Notably, while CM is recognized as a crucial mediator of sperm transport, survival, and fertilization, its molecular makeup has not been systematically studied in Bali cattle or other native Indonesian breeds. Earlier research has primarily evaluated CM based on biophysical traits such as appearance, viscosity, spinnbarkeit, and crystallization patterns, which are useful but provide only indirect, descriptive data on fertility. The lack of molecular-level information hampers accurate distinction between fertile and subfertile females in field conditions.

Furthermore, most proteomic studies in livestock reproduction have focused on male reproductive fluids, such as seminal plasma and spermatozoa, with relatively few investigations of female reproductive secretions. Even when CM proteomics has been examined in other species, the results cannot be easily applied to Bali cattle due to breed-specific genetics, adaptive physiology, and tropical production environments. Crucially, no studies have yet combined biophysical assessment of CM with high-resolution proteomic profiling to find fertility-related biomarkers in Bali heifers. As a result, molecular markers that could aid objective fertility assessment and improve the success of AI in this breed remain unknown. This gap hinders the development of evidence-based, biomarker-guided selection strategies for breeding females and limits the optimization of AI programs in Bali cattle.

In response to these gaps, the present study aims to thoroughly characterize the proteomic profile of CM in Bali heifers at oestrus and to identify molecular signatures linked to fertility-related mucus quality. Specifically, this study seeks to (i) categorize CM into good- and poor-quality groups using a standardized five-parameter biophysical scoring system, (ii) profile and compare the CM proteome between these groups using liquid chromatography–tandem mass spectrometry (LC–MS/MS), (iii) identify proteins with different abundances and their related biological pathways associated with either favorable or unfavorable fertility traits, and (iv) assess the potential of candidate proteins as biomarkers through correlation and receiver-operating-characteristic analyses. By combining phenotypic assessment with proteomic and bioinformatic analyses, this study aims to create a molecular framework for fertility evaluation and support the development of biomarker-based strategies to improve AI efficiency in Bali cattle.

## MATERIALS AND METHODS

### Ethical approval

All procedures involving animals in this study were performed in strict accordance with internationally accepted guidelines for animal care and use in research and adhered to national and institutional regulations on animal welfare. The study protocol, which includes animal selection, handling, CM collection, and laboratory procedures, was reviewed and officially approved by the Animal Ethics Committee of the National Research and Innovation Agency (BRIN), Indonesia (Approval Certificate No. 050/KE.02/SK/03/2023).

Before starting the study, ethical approval was secured to ensure that all procedures minimized stress, pain, and discomfort for the animals. Only clinically healthy Bali heifers were included, and they were monitored throughout the study for any signs of distress or health issues. CM sampling was done aseptically during natural oestrus using non-invasive methods and standard AI equipment, without hormonal synchronization or surgical procedures.

Animal handling and sample collection were conducted by trained personnel experienced in bovine reproductive management to ensure animal welfare and procedural consistency. The study followed the principles of the 3Rs (Replacement, Reduction, and Refinement) by limiting the number of animals to the minimum necessary for statistical validity, using non-terminal sampling methods, and refining procedures to reduce handling time and discomfort.

No animals were harmed, euthanized, or subjected to experimental treatments beyond routine reproductive management practices. All animals were returned to normal herd management immediately after sample collection. The study was conducted in accordance with the ethical standards outlined in the Animal Research: Reporting of *In Vivo* Experiments 2.0 guidelines for reporting animal research and relevant national animal welfare laws in Indonesia.

### Study period and location

This study was conducted from February to August 2024 in Lappariaja District, Bone Regency, South Sulawesi, Indonesia (4°27′12′′ S, 120°5′18′′ E). The study area features a tropical monsoon climate with average temperatures ranging from 27°C to 30°C and relative humidity between 70% and 85%. Sample preparation and routine laboratory analyses were performed at the Laboratory of Animal Reproduction, Faculty of Animal Science, Hasanuddin University, Makassar, Indonesia. Proteomic profiling was conducted at the Zoological Research Center, National Research and Innovation Agency (BRIN), Cibinong, West Java, Indonesia.

### Experimental animals and management conditions

A total of 40 purebred Bali heifers aged 2–3 years were enrolled in this study. All animals were healthy and free of reproductive and infectious diseases, as confirmed by veterinary examinations and vaccination records prior to inclusion. The heifers were kept under a semi-intensive management system, grazing daily on natural grass pastures dominated by Brachiaria decumbens and Paspalum conjugatum. Shaded resting areas and clean drinking water were provided *ad libitum* and refreshed twice-daily. Additionally, each animal received a concentrate supplement containing 12%–14% crude protein at approximately 1% of body weight per day. Oestrus detection was performed by trained personnel through twice-daily visual observations (morning and evening), based on behavioral signs such as vulvar swelling, mucus discharge, restlessness, and mounting behavior. No hormonal synchronization was used to ensure evaluation under natural oestrus conditions.

### CM collection and handling

CM samples were aseptically collected from each heifer during the oestrus phase, within 5–30 min after oestrus detection. Collection was performed using a sterile AI sheath attached to a 10 mL syringe under vaginal speculum guidance. To minimize inter-observer variation, all collections were carried out by the same trained technician. Immediately after collection, mucus samples were transferred into sterile 2-mL microtubes, properly labeled, and kept on ice (4°C) during transportation to the laboratory, where processing was completed within 2 h.

### Biophysical evaluation and classification of CM

CM quality was evaluated using a five-parameter biophysical scoring system adapted from Diansyah *et al*. [9]. The parameters assessed included visual appearance, viscosity, spinnbarkeit (stretchability), crystallization pattern (fern test), and pH. The pH was measured with a calibrated digital micro-pH meter (Hanna Instruments, USA). Each parameter received a score from 1 to 3, leading to a maximum total score of 15. Samples with scores above 7 were classified as good CM (GCM), while those with scores of 7 or less were classified as poor CM (PCM). Scoring was conducted independently by two trained observers, and inter-observer reliability was confirmed with Cohen’s kappa coefficient (κ = 0.89), indicating excellent agreement.

### Protein extraction and quantification

Protein extraction from CM samples was performed prior to electrophoretic separation following a modified protocol described by Diansyah *et al*. [10]. Because the original protocol was optimized for semen samples, it was adapted for CM by incorporating a stronger detergent-based lysis buffer (1% SDS) and maintaining cold processing (4°C) throughout to improve solubilization of mucus-associated proteins and minimize proteolytic degradation. To prevent protein degradation, each sample was homogenized in lysis buffer containing 50 mM Tris-HCl (pH 7.5), 150 mM NaCl, 1% SDS, and a protease inhibitor cocktail (Sigma-Aldrich, USA). Homogenates were vortexed and centrifuged at 12,000 × *g* for 15 min at 4°C, and the resulting supernatant containing soluble proteins was collected. Protein concentration was determined using the Bradford method with bovine serum albumin as the standard, and absorbance was measured at 595 nm using a UV–Vis spectrophotometer (Thermo Scientific, USA). The average protein yield ranged from 1.8 to 3.2 mg/mL. All measurements were conducted in five technical replicates to ensure analytical precision and reproducibility.

### Sodium dodecyl sulfate–polyacrylamide gel electrophoresis (SDS–PAGE) separation and gel processing

Protein separation was performed using one-dimensional SDS–PAGE (1D SDS–PAGE) according to Diansyah *et al*. [10], with minor modifications. Since the original method was designed for semen-derived proteins, sample preparation and electrophoresis conditions were slightly adjusted for CM proteins to improve band resolution and reduce smearing typically associated with highly viscous, mucin-rich samples. Gels comprised a 12% resolving gel and a 4% stacking gel cast in a Mini-PROTEAN® electrophoresis system (Bio-Rad, USA). Samples were mixed with loading buffer, heated at 95 °C for 5 min, and loaded alongside a broad-range molecular weight marker (ExcelBand PM2700; SMOBIO). Electrophoresis was conducted at 60 V for 30 min followed by 120 V for 3 h. Gels were stained with 0.1% Coomassie Brilliant Blue R-250 for 1 h and destained using 40% methanol and 10% acetic acid until optimal contrast was achieved. Protein bands were documented, compared with molecular weight markers, and visually distinct bands were excised for downstream LC–MS/MS analysis.

### In-gel digestion and peptide preparation

Excised protein bands underwent in-gel digestion following the modified protocol of Diansyah *et al*. [10]. As the original protocol was developed for semen-derived protein bands, it was adapted for CM by applying the same in-gel digestion workflow with adjustments in the destaining and peptide extraction steps to accommodate the mucus-derived protein matrix. Gel pieces were destained, dehydrated, reduced with tris(2-carboxyethyl) phosphine, alkylated with iodoacetamide, and rehydrated with trypsin solution (10 ng/μL; Promega, USA). Digestion occurred at 37°C for 4 h. Peptides were extracted using 70% acetonitrile with 0.1% trifluoroacetic acid, vacuum-dried, and reconstituted in 2% acetonitrile with 0.1% formic acid. All peptide samples were stored at −20°C until LC–MS/MS analysis.

### LC–MS/MS analysis

Peptide analysis was conducted using a Nano LC Ultimate 3000 system connected to a Q Exactive Plus Orbitrap mass spectrometer (Thermo Scientific) as described by Diansyah *et al*. [10]. Peptides were separated on an Acclaim PepMap 100 C18 nano-column at a flow rate of 300 nL/min with a 90-min linear gradient. Data were collected in positive ion mode through data-dependent acquisition, selecting the top 10 precursor ions for fragmentation. Mass spectra were captured at resolutions of 70,000 (MS1) and 17,500 (MS2), with a dynamic exclusion period of 30 s.

### Protein identification and data processing

Raw LC–MS/MS data were analyzed using Proteome Discoverer v2.2 with the Sequest HT search algorithm against the UniProt *Bos taurus* proteome database (release 2024_02). Trypsin specificity with up to two missed cleavages was applied, and mass tolerances were set at ±10 ppm for precursors and 0.02 Da for fragments. Protein identification was validated at a 1% false discovery rate (FDR) using a target-decoy strategy. Quantitative data were log10-transformed, median-centered, and missing values were imputed using k-nearest neighbors. The processed proteomic dataset was deposited in the PRIDE repository under accession number PXD045672 (https://www.uniprot.org) [11].

### Statistical analysis

The CM quality data were analyzed using IBM SPSS Statistics v26.0 (IBM Corp., NY, USA). Normality and homogeneity of variance were checked with Shapiro–Wilk and Levene’s tests, respectively. Group comparisons between GCM and PCM were conducted using independent-samples t-tests or Mann–Whitney U tests, as appropriate. Proteomic data analysis and visualization were performed in MetaboAnalyst using Benjamini–Hochberg FDR correction (q < 0.05). Functional enrichment analysis was performed with g:Profiler (g:GOSt) (https://biit.cs.ut.ee/gprofiler/) based on the Gene Ontology database (release 2024_03). A Pearson correlation analysis of CM quality traits and protein levels was performed in Python v3.10, with visualizations created using Matplotlib v3.8.0 and Seaborn v0.12.2.

## RESULTS

### Quality assessment of CM

As summarized in Table 1, statistical analysis showed significant differences (p < 0.05) between the GCM and PCM groups for four biophysical parameters: appearance, viscosity, spinnbarkeit, and ferning pattern. In these parameters, the GCM group consistently had higher average scores, indicating better physical and functional mucus qualities. Conversely, there was no significant difference in pH values between the two groups. This suggests that, within this population, pH does not serve as a primary indicator of CM quality but may act as a covariate or modulatory factor.

**Table 1:** Cohort and quality of cervical mucus in Bali heifers.

Group	N	Appearance	Viscosity	Spinnbarkeit	Fern pattern	pH
GCM	20	2.6 ± 0.55^a^	2.2 ± 0.45^a^	2.2 ± 0.84^a^	2.2 ± 0.84^a^	2.0 ± 0.71^a^
PCM	20	1.2 ± 0.45^b^	1.40 ± 0.89^b^	1.2 ± 0.45^b^	1.4 ± 0.55^b^	1.60 ± 0.55^a^

GCM = Good cervical mucus, PCM = Poor cervical mucus, N = Number of animals. Values are presented as mean ± standard deviation. Different superscript letters (a, b) within the same column indicate statistically significant differences (*p* < 0.05).

### Protein identification and distribution patterns

**Figure 1:**
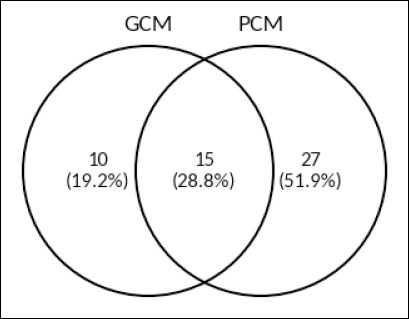
Venn diagram showing the distribution of cervical mucus proteins retained after quality control in good cervical mucus (GCM) and poor cervical mucus (PCM) groups. Shared and group-specific proteins identified following liquid chromatography–tandem mass spectrometry analysis are indicated.

After quality control (QC) filtering, 52 unique proteins were retained for further analysis (Figure 1). Among these, 15 proteins (28.8%) were found in both the GCM and PCM groups, while 10 proteins (19.2%) were exclusive to GCM, and 27 proteins (51.9%) were unique to PCM. The final set of proteins after QC included 25 in GCM and 42 in PCM. This uneven distribution suggests a more extensive and complex proteomic profile in PCM, which was further examined through differential abundance and functional analysis.

### Global proteomic separation between GCM and PCM

**Figure 2:**
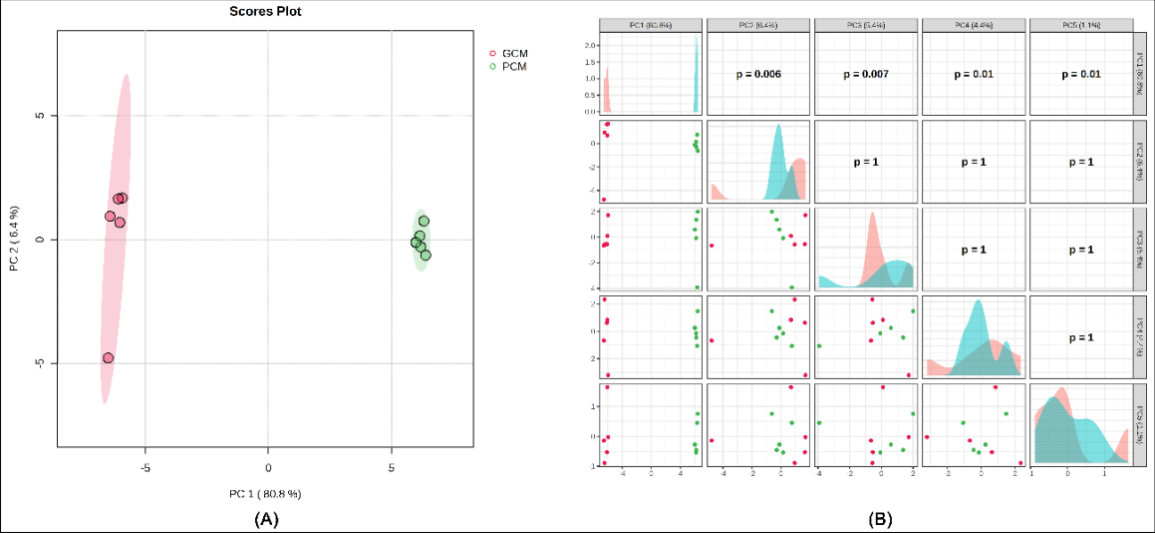
Principal component analysis (PCA) of cervical mucus proteins. (A) PCA scores plot showing separation between good cervical mucus (GCM) and poor cervical mucus (PCM) along principal component 1 (PC1, 80.8% variance explained) and principal component 2 (PC2, 6.4%), with 95% confidence ellipses. (B) Pairwise scatter and density plots of principal components with corresponding group comparison p-values.

Principal component analysis (PCA) showed a clear separation between the GCM and PCM groups (Figure 2). The group separation was mainly driven by the principal component 1 (PC1), which explained 80.8% of the total variance, with some variation along PC2 (6.4%). Pairwise comparisons of principal components (Figure 2A) confirmed that the main components differed significantly between groups (p < 0.05), while later components showed no significant separation. The PCA scores plot (Figure 2B) displayed two tight, non-overlapping clusters representing GCM and PCM, with no overlap of the 95% confidence ellipses. These findings indicate a strong overall proteomic difference between GCM and PCM.

### Differentially abundant proteins and expression profiles

**Figure 3:**
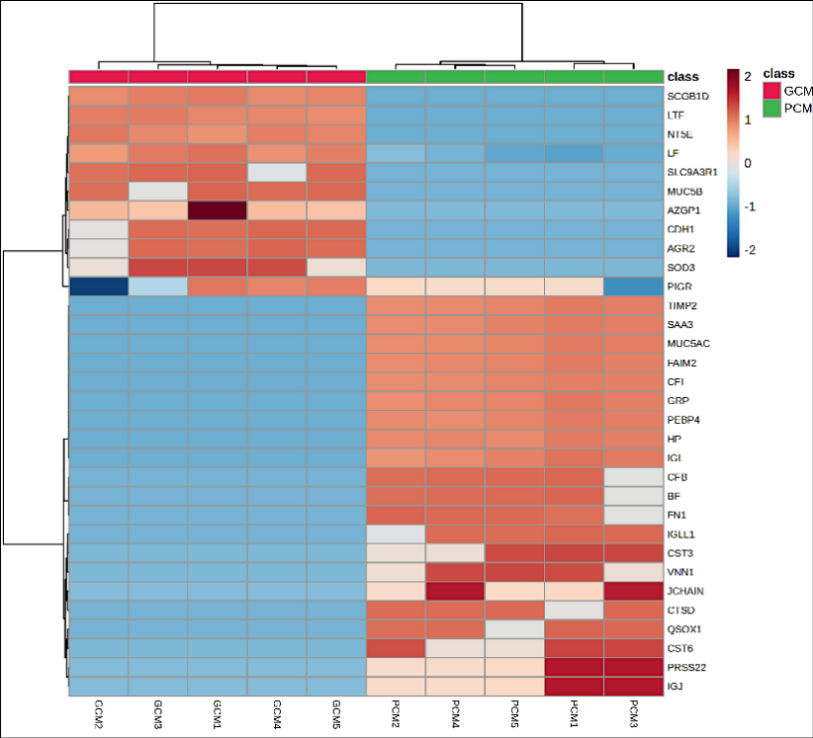
Hierarchical clustering heatmap of discriminative cervical mucus proteins showing distinct expression patterns between good cervical mucus (GCM) and poor cervical mucus (PCM) groups. Protein abundance was scaled across samples, and clustering reveals clear group-wise separation.

**Figure 4:**
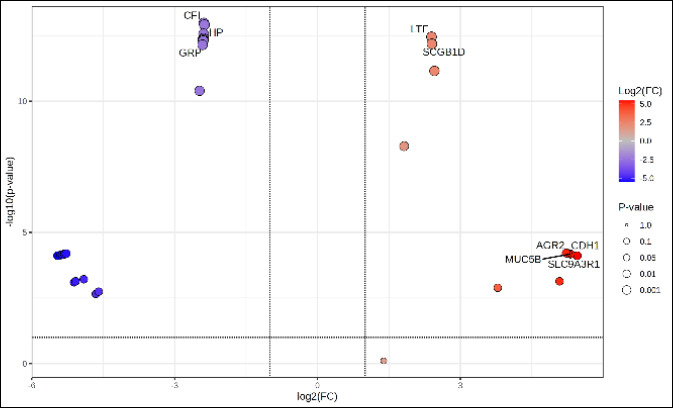
Volcano plot of differentially expressed cervical mucus proteins between good cervical mucus and poor cervical mucus groups.

**Figure 5:**
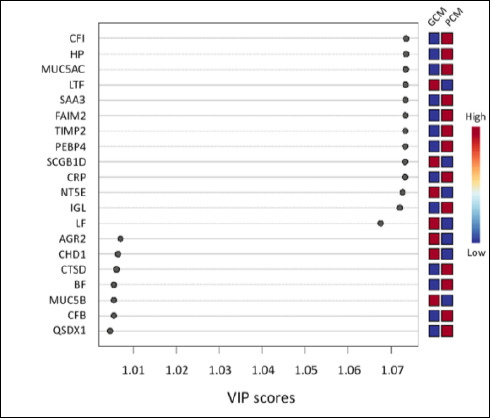
Partial least squares–discriminant analysis (PLS-DA) variable importance in projection (VIP) scores of the top 20 cervical mucus proteins contributing to discrimination between good cervical mucus (GCM) and poor cervical mucus (PCM) groups.

Hierarchical clustering heatmap analysis (Figure 3) revealed a clear grouping of samples that matched their clinical classification. The GCM group showed higher levels of Ecto-5′-nucleotidase (NT5E) (CD73), LTF, SCGB1D, and LF, whereas PCM samples showed enrichment in MUC5AC, FAIM2, Complement factor I (CFI), Haptoglobin (HP), TIMP2, PEBP4, SAA3, GRP, and IGL. Consistent with these results, the volcano plot (Figure 4) highlighted PCM-dominant proteins, especially CFI, HP, MUC5AC, and GRP, as showing the largest and most statistically significant negative changes. Conversely, NT5E, LTF, and SCGB1D exhibited significant positive changes favoring GCM. A full summary of effect sizes, p-values, and expression directions is provided in Table 2, confirming these proteins as the strongest discriminators between groups. Additionally, the top-20 features contributing to group separation (Figure 5) again ranked CFI, HP, and MUC5AC highest, while also identifying LTF, FAIM2, TIMP2, and PEBP4 as additional key markers distinguishing GCM from PCM.

**Table 2:** Proteins differentially expressed between GCM and PCM.

Gene ID	FC	log2(FC)	p-value	–log10(p)	Direction
CFI	19.144	–23.851	1.05E-09	12.977	PCM
HP	19.273	–23.753	1.23E-09	12.91	PCM
MUC5AC	19.032	–23.935	2.74E-10	12.563	PCM
LTF	5.254	2.393	3.57E-09	12.448	GCM
SAA3	19.017	–23.946	4.06E-09	12.391	PCM
FAIM2	18.894	–2.404	4.34E-10	12.362	PCM
TIMP2	1.895	–23.998	4.65E-09	12.332	PCM
PEBP4	18.945	–24.001	5.03E-09	12.298	PCM
SCGB1D	5.282	2.401	6.56E-09	12.183	GCM
GRP	18.749	–24.151	7.13E-09	12.147	PCM
NT5E	5.483	2.455	7.03E-09	11.153	GCM
IGL	17.956	–24.775	4.03E-08	10.394	PCM
LF	3.525	1.818	5.18E-05	8.2854	GCM
AGR2	37.308	5.221	6.08E-01	4.2158	GCM
CDH1	38.186	5.255	6.30E-01	4.2005	GCM
CTSD	25.817	–52.755	6.43E-01	4.1919	PCM
BF	25.207	–5.31	6.66E-01	4.1766	PCM
MUC5B	39.701	5.311	6.66E-01	4.1764	GCM
CFB	25.185	–53.113	6.67E-02	4.1761	PCM
QSOX1	24.407	–53.566	7.00E-01	4.1548	PCM
FN1	23.835	–53.908	7.25E-02	4.1394	PCM
IGLL1	22.762	-54.572	7.70E-01	4.1133	PCM
SLC9A3R1	43.821	5.454	7.75E-02	4.1106	GCM
VNN1	33.306	–49.081	61.221	3.2131	PCM
CST6	29.564	–5.08	73.007	3.1366	PCM
SOD3	33.882	5.082	73.138	3.1359	GCM
CST3	2.891	–51.123	7.977	3.0982	PCM
AZGP1	13.763	3.783	12.906	2.8892	GCM
JCHAIN	41.364	–45.955	18.148	2.7412	PCM
IGJ	39.747	–4.653	22.039	2.6568	PCM
PRSS22	39.674	–46.557	22.111	2.6554	PCM

GCM = Good cervical mucus, PCM = Poor cervical mucus, FC = Fold change, log2(FC) = Log2-transformed fold change, −log10(p) = Negative log10-transformed p-value. Differential expression was determined using false discovery rate–adjusted statistics, and proteins were classified according to their direction of abundance in GCM or PCM.

### Functional enrichment analysis

**Figure 6:**
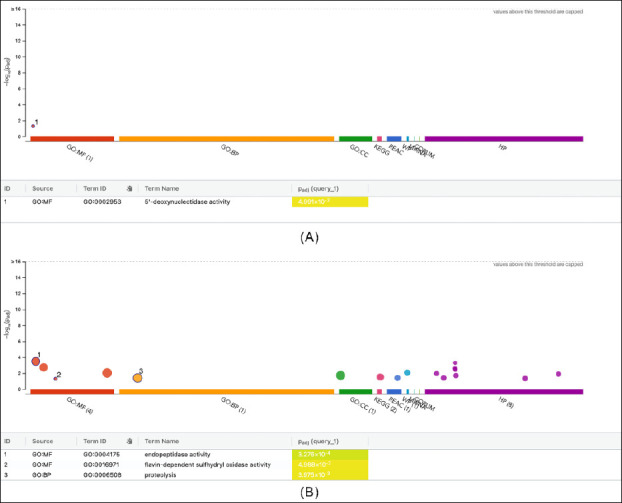
Functional enrichment analysis of direction-specific cervical mucus protein sets. Enriched Gene Ontology terms associated with (A) good cervical mucus (GCM) and (B) poor cervical mucus (PCM) are shown based on differentially abundant proteins.

Functional enrichment analysis identified distinct biological signatures linked to CM quality (Figure 6). The GCM-associated protein set showed a single significant term, 5′-deoxynucleotidase activity (GO:0002953), driven by NT5E (CD73), which aligns with extracellular adenosine production and a more regulated, anti-inflammatory microenvironment. In contrast, the PCM-associated protein set was enriched for endopeptidase and proteolysis activities, primarily driven by CFI and HP, along with flavin-dependent sulfhydryl oxidase activity involving QSOX1. These functions indicate heightened complement turnover, protein processing, and disulfide-bond formation, characteristic of extracellular matrix (ECM) remodeling and increased viscosity in poor-quality CM. Overall, these functional signatures reinforce NT5E as a marker of GCM, while CFI, HP, and QSOX1 suggest PCM.

### Biomarker performance and classification accuracy

**Figure 7:**
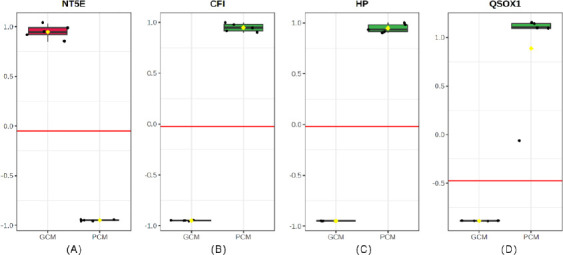
Receiver-operating characteristic curves illustrating the classification performance of candidate protein biomarkers distinguishing good cervical mucus (GCM) from poor cervical mucus (PCM): (A) NT5E, (B) CFI, (C) haptoglobin (HP), and (D) QSOX1. Area under the curve (AUC) values indicate discriminatory accuracy.

Single-protein receiver-operating characteristic analysis showed complete separation of groups in this cohort. NT5E, elevated in GCM, showed an apparent area under the curve (AUC) of 1.00, with perfect sensitivity and specificity at the optimal cutoff (Figure 7A; Tables 3 and 4). Similarly, the PCM-enriched proteins CFI and HP each achieved an AUC of 1.00 with perfect classification performance (Figures 7B and 7C), consistent with their strong negative log_2_ fold changes and highly significant p-values. QSOX1 also demonstrated perfect separation (Figure 7D); however, its differential abundance was not statistically significant (p = 0.70), suggesting that this marker should be considered exploratory until independently validated. Overall, NT5E emerged as the leading GCM-related biomarker candidate, while CFI and HP were identified as the primary PCM-associated markers, with classification thresholds set by the maximum Youden index (Table 4). Notably, NT5E, CFI, and HP have not been previously reported in bovine CM, thus representing newly identified molecular markers linked to fertility-related mucus quality.

**Table 3:** Functional annotation of selected proteins and implications for quality of cervical mucus.

Term name	Term ID	Direction	Gene ID	Biological function	Implication
5’-deoxynucleotidase activity	GO:0002953	GCM	NT5E	Ecto-5′-nucleotidase hydrolyzes extracellular AMP to adenosine, dampening inflammation and supporting epithelial barrier function.	Favorable (GCM) - consistent with an anti-inflammatory, well-regulated milieu.
Endopeptidase activity	GO:0004175	PCM	CFI	Plasma serine protease inactivates C3b and C4b with cofactors (CFH, C4BP, CR1/MCP), thereby constraining complement activation.	Unfavorable (PCM) - suggests heightened complement turnover/immune activation.
Flavin-dependent sulfhydryl oxidase activity	GO:0016971	PCM	QSOX1	FAD-dependent sulfhydryl oxidase in the secretory pathway that catalyzes disulfide-bond formation in secreted/ECM proteins, generating H_2_O_2_.	Unfavorable (PCM) - may indicate increased matrix cross-linking/viscosity.
Proteolysis	GO:0006508	PCM	HP	Acute-phase glycoprotein that binds free hemoglobin for clearance by CD163^+^ macrophages, limiting oxidative stress and iron loss.	Unfavorable (PCM) - marker of inflammatory/oxidative stress responses

GCM = Good cervical mucus, PCM = Poor cervical mucus, GO = Gene ontology, AMP = Adenosine monophosphate, FAD = Flavin adenine dinucleotide, ECM = Extracellular matrix, H_2_O_2_ = Hydrogen peroxide. Functional terms were assigned based on Gene Ontology enrichment, and direction indicates association with GCM or PCM.

**Table 4:** Classification performance of candidate biomarkers.

Gene ID	Direction	AUC	Cutoff	Sensitivity	Specificity	log2FC	p-value
NT5E	GCM	1.0	–0.0504632	1.0	1.0	2.455	7.03E-09
CFI	PCM	1.0	–0.0248054	1.0	1.0	–2.385	1.05E-09
HP	PCM	1.0	–0.0210049	1.0	1.0	–2.375	1.23E-09
QSOX1	PCM	1.0	–0.473473	1.0	1.0	–5.357	7.00E-01

GCM = Good cervical mucus, PCM = Poor cervical mucus, AUC = Area under the receiver-operating characteristic curve, log2FC = Log2-transformed fold change. Cutoff values were determined using the maximum Youden index. Sensitivity and specificity are expressed as proportions, and p-values indicate statistical significance of differential protein abundance.

### Correlation between CM traits and protein biomarkers

**Figure 8:**
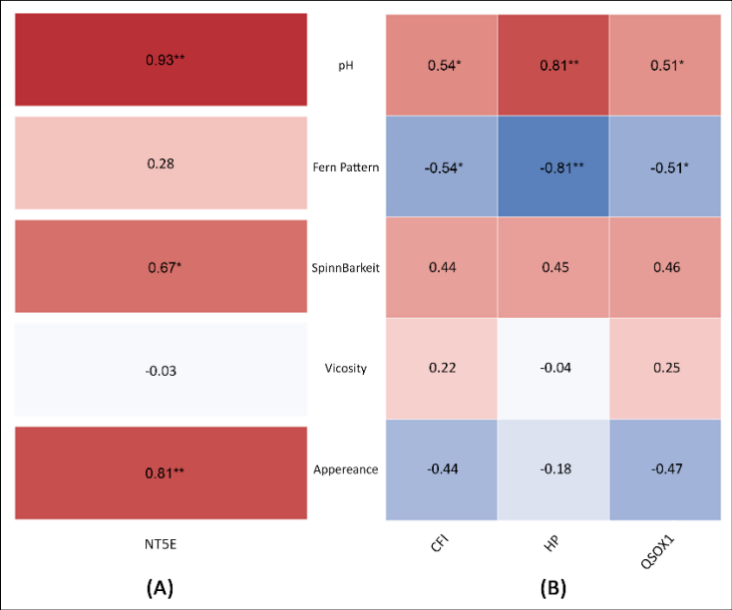
Pearson correlation heatmap showing relationships between cervical mucus quality parameters and protein abundance. Correlations are presented separately for (A) good cervical mucus (GCM) and (B) poor cervical mucus (PCM). Positive and negative correlations are indicated in red and blue, respectively; * and ** denote moderate and strong correlations with statistical significance (p < 0.05).

Pearson correlation analysis between CM quality parameters and protein abundance is shown separately for GCM (Figure 8A) and PCM (Figure 8B). In the GCM group, NT5E displayed a strong positive correlation with pH (r = 0.93) and appearance (r = 0.81), as well as a moderate positive correlation with spinnbarkeit (r = 0.67). Conversely, NT5E showed no significant relationship with viscosity (r = 0.03) and only a weak correlation with the ferning pattern (r = 0.28).

In the PCM group, CFI, HP, and QSOX1 were consistently and positively associated with pH, with correlation coefficients of 0.54, 0.81, and 0.51, respectively. Conversely, all three proteins were negatively correlated with the ferning pattern, with coefficients ranging from 0.54 to 0.81. Spinnbarkeit showed positive correlations with all three proteins (r = 0.44–0.48), while viscosity exhibited weak or negligible associations (r = 0.04–0.25). Notably, appearance was negatively correlated with QSOX1 (r = 0.47).

Overall, these results show specific and group-related relationships between CM quality traits and protein levels. Strong links were mainly observed between pH and the ferning pattern, while viscosity did not show consistent or significant associations with the evaluated protein biomarkers.

## DISCUSSION

### Overview of CM proteomic characterization

This study offers the first detailed analysis of the CM proteome in Bali heifers and identifies protein factors that differentiate GCM from PCM. CM is essential for fertility because it acts as the biochemical and structural environment through which sperm must travel to reach the oocyte [3, 9]. Past research in both bovine and human reproduction has shown a strong link between CM quality and the chances of conception, with positive biophysical and biochemical traits enhancing sperm survival and movement [12, 13]. By combining phenotypic assessment, proteomic analysis (Figures 1–5), functional enrichment, biomarker assessment, and correlation studies, this research deepens our understanding of the molecular factors that influence CM quality in an indigenous cattle breed.

### Biophysical characteristics of CM and fertility relevance

Assessment of CM quality parameters revealed significant differences between GCM and PCM groups in appearance, viscosity, spinnbarkeit, and ferning pattern, while pH did not differ significantly. The superior physical attributes of GCM observed in this study align with previous reports indicating that transparent, elastic, and well-crystallized mucus promotes sperm penetration and successful fertilization [9, 14]. In contrast, PCM was characterized by turbidity, decreased elasticity, and weak crystallization patterns, all of which are known to hinder sperm migration and lower fertilization efficiency [15]. These phenotypic differences provided a solid basis for subsequent proteomic comparisons.

### Proteomic landscape and group-specific molecular signatures

After QC, 52 unique proteins were identified, including 10 proteins exclusive to GCM, 27 exclusive to PCM, and 15 shared between groups. The broader proteomic repertoire observed in PCM indicates a more complex yet dysregulated molecular environment, consistent with immune activation and oxidative stress previously reported in subfertile reproductive fluids [16]. PCA and hierarchical clustering heatmaps showed complete separation between GCM and PCM samples, providing strong evidence for distinct molecular signatures associated with CM quality.

### Differentially abundant proteins and biological interpretation

Differential abundance analysis showed that NT5E, LTF, SCGB1D, and LF were significantly enriched in GCM, while PCM exhibited higher levels of CFI, HP, MUC5AC, FAIM2, TIMP2, PEBP4, SAA3, GRP, and IGL. Proteins enriched in GCM mainly serve protective and regulatory roles. NT5E catalyzes the conversion of adenosine monophosphate to adenosine, promoting anti-inflammatory signaling and maintaining epithelial barrier integrity [17]. LTF and LF are iron-binding glycoproteins that help defend against microbes and reduce oxidative stress within the reproductive tract [18, 19]. Conversely, proteins enriched in PCM are mostly linked to inflammatory responses and ECM remodeling. CFI controls complement activation and indicates increased immune activity [20], whereas HP is an acute-phase protein associated with oxidative stress and inflammation [21]. Overexpression of MUC5AC has been linked to increased mucus viscosity and impaired sperm transport [12], while QSOX1 promotes disulfide-bond formation, which may stiffen the ECM [22].

### Multivariate ranking and pathway-level insights

Partial least squares–discriminant analysis further highlighted CFI, HP, and MUC5AC as dominant PCM-associated proteins, while NT5E and LTF emerged as key GCM markers. These findings suggest that CM quality depends not only on the abundance of structural glycoproteins but also on the balance between immune activation, oxidative stress, and antioxidant defense mechanisms.

### Functional enrichment and mechanistic implications

Functional enrichment analysis showed that GCM was specifically enriched for 5′-deoxynucleotidase activity mediated by NT5E, supporting the role of extracellular adenosine in maintaining an anti-inflammatory and homeostatic mucosal environment [23]. In contrast, PCM was enriched for proteolytic activity (HP), complement regulation (CFI), and flavin-dependent sulfhydryl oxidase activity (QSOX1). These pathways collectively indicate immune activation, oxidative stress, and structural cross-linking, all of which hinder sperm transport and align with the unfavorable physical properties observed in PCM. Similar dysregulation of complement and oxidative pathways has been reported in human reproductive fluids and has been linked to subfertility [12, 16].

### Biomarker performance and fertility prediction potential

The evaluation of biomarker classification performance showed that NT5E, CFI, and HP achieved perfect discrimination between GCM and PCM (AUC = 1.0). NT5E was a favorable biomarker of high-quality mucus, whereas CFI and HP consistently indicated poor-quality mucus. Although QSOX1 also demonstrated group separation, its lack of statistical robustness suggests it should be considered exploratory until validated independently. These findings align with bovine seminal plasma studies reporting associations among complement inhibitors, acute-phase proteins, and impaired sperm function [24]. Mechanistically, NT5E-derived adenosine likely reduces epithelial inflammation and stabilizes mucosal barrier properties, promoting low viscosity, high elasticity, and optimal ferning patterns. Conversely, increased activity of CFI, HP, and QSOX1 reflects an inflammatory and oxidative microenvironment that stiffens the mucus matrix and impairs sperm penetrability [25].

### Correlation between protein biomarkers and mucus quality traits

Pearson correlation analysis further clarified the relationships between protein biomarkers and CM quality parameters. In GCM, NT5E showed strong positive correlations with pH and appearance and a moderate correlation with spinnbarkeit, highlighting its key role in maintaining favorable mucus characteristics. In PCM, CFI, HP, and QSOX1 were positively correlated with pH but negatively correlated with the ferning pattern, indicating disturbances in mucus crystallization. These findings agree with functional enrichment results that emphasize complement turnover and oxidative remodeling in PCM. Notably, viscosity showed little correlation with protein abundance, supporting previous evidence that viscosity is more affected by mucin glycosylation and ionic composition than by total protein levels [26]. Similar enrichment of complement and redox pathways has been reported across species, including humans and small tropical ruminants, demonstrating the broader biological importance of these mechanisms [27].

### Study limitations and future directions

Several limitations should be recognized. The relatively small cohort size requires cautious interpretation of biomarker performance and calls for validation in larger, independent populations. The untargeted proteomic approach favored abundant proteins and may have overlooked low-abundance signaling molecules, such as cytokines and steroid-binding proteins. Moreover, although sampling was standardized at oestrus, endocrine parameters (e.g., estradiol and progesterone) and environmental factors (e.g., heat stress, nutritional status, and subclinical inflammation) were not measured and could influence the CM proteome. The cross-sectional design also prevented direct assessment of conception outcomes. Future studies should incorporate hormonal and environmental measurements, use targeted validation methods (e.g., ELISA or PRM), and prospectively connect biomarker profiles to pregnancy outcomes following AI.

### Implications for reproductive management

Despite these limitations, the findings offer important mechanistic and practical insights. The results emphasize the critical role of anti-inflammatory and antioxidant pathways in favorable CM, and identify immune activation and oxidative stress as signs of poor-quality CM. From a practical standpoint, NT5E, CFI, and HP are promising biomarkers for evaluating fertility potential in Bali heifers, with potential applications for improving AI candidate selection and reproductive success. Additionally, the combined approach of phenotypic assessment, proteomics, bioinformatics, and correlation analysis provides a reliable model for biomarker discovery in livestock reproduction.

## CONCLUSION

This study presents the first comprehensive molecular evidence linking the composition of CM proteome with fertility-related mucus quality in Bali heifers. By combining biophysical assessments with high-resolution LC–MS/MS–based proteomic profiling, clear and consistent differences were observed between GCM and PCM. GCM was characterized by superior physical qualities, including appearance, viscosity, spinnbarkeit, and ferning pattern, while pH showed no significant difference between the groups. Proteomic analysis identified 52 proteins after QC, of which 13 showed significant differences in abundance. Notably, NT5E, LTF, SCGB1D, and LF were enriched in GCM, whereas PCM was dominated by CFI, HP, MUC5AC, FAIM2, TIMP2, PEBP4, SAA3, GRP, and IGL, reflecting distinct biological states associated with fertility potential.

Functionally, GCM was associated with anti-inflammatory and epithelial-protective mechanisms, especially through NT5E-mediated extracellular adenosine production, while PCM showed signs of complement activation, proteolysis, oxidative stress, and ECM remodeling. Biomarker analysis demonstrated excellent discriminative ability for NT5E, CFI, and HP (AUC = 1.0), underscoring their potential as molecular indicators of favorable or unfavorable CM quality. Correlation analyses further supported these findings by revealing strong links between key protein markers and specific mucus quality traits, particularly appearance, ferning pattern, and pH.

A major strength of this study is its comprehensive methodological approach, which integrates standardized biophysical scoring, untargeted proteomics, multivariate analysis, functional enrichment, and biomarker validation within one experimental design. Focusing on Bali cattle, an indigenous breed of significant socio-economic importance, further enhances the relevance and novelty of the findings, especially given that similar molecular data on female reproductive secretions in tropical livestock remain limited.

In conclusion, the current study identifies CM proteomic signatures as reliable molecular indicators of fertility-related mucus quality in Bali heifers. NT5E, CFI, and HP stand out as promising candidate biomarkers that could support objective fertility assessments and enhance AI decision-making. These findings establish a molecular basis for developing biomarker-assisted reproductive management strategies and offer a reproducible model for advancing fertility research in indigenous and underrepresented livestock populations.

## DATA AVAILABILITY

The supplementary data can be made available from the corresponding author upon request.

## AUTHORS’ CONTRIBUTIONS

MY, ALT, HH, AB, AMD, SSa, MM, and SSh: Conceived, designed, and coordinated the study. AMD, MM, HH, SSh, AFSA, RR, and AMA: Contributed and served as the principal investigators. MY, AMD, AB, and SSa: Designed data collection tools. AMD, MM, HH, AMA, and MJ: Supervised field sampling, data collection, laboratory work, and data entry. AMD, AMA, RR, and MJ.: Performed statistical analysis, interpretation, and drafted the manuscript. All authors have read and approved the final version of the manuscript.
